# The Redox architecture of physiological function

**DOI:** 10.1016/j.cophys.2019.04.009

**Published:** 2019-06

**Authors:** Jerome Santolini, Stephen A Wootton, Alan A Jackson, Martin Feelisch

**Affiliations:** 1 Institute for Integrative Biology of the Cell (I2BC), CEA, CNRS, Univ Paris-Sud, Universite Paris-Saclay, F-91198, Gif-sur-Yvette Cedex, France; 2 Human Nutrition, University of Southampton and University Hospital Southampton, Tremona Road, Southampton, SO16 6YD, UK; 3 Clinical and Experimental Sciences, Faculty of Medicine and Institute for Life Sciences, University of Southampton, NIHR Southampton Biomedical Research Centre, Southampton General Hospital, Tremona Road, Southampton, SO16 6YD, UK

## Abstract

The ability of organisms to accommodate variations in metabolic need and environmental conditions is essential for their survival. However, an explanation is lacking as to how the necessary accommodations in response to these challenges are organized and coordinated from (sub)cellular to higher-level physiological functions, especially in mammals. We propose that the chemistry that enables coordination and synchronization of these processes dates to the origins of Life. We offer a conceptual framework based upon the nature of electron exchange (Redox) processes that co-evolved with biological complexification, giving rise to a multi-layered system in which intra/intercellular and inter-organ exchange processes essential to sensing and adaptation stay fully synchronized. Our analysis explains why Redox is both the *lingua franca* and the *mechanism* that enable integration by connecting the various elements of regulatory processes. We here define these interactions across levels of organization as the ‘*Redox Interactome*’. This framework provides novel insight into the chemical and biological basis of Redox signalling and may explain the recent convergence of metabolism, bioenergetics, and inflammation as well as the relationship between Redox stress and human disease.

## Highlights

• Organisms need to adapt to changes in metabolic demand/environmental conditions to survive.
• Those adaptations require coordinated adjustments in energy utilization, mitochondrial function, and intermediary metabolism.
• Electron exchange processes are central to enabling integrated adjustments across multiple levels of organization.
• The fundamental chemistry of this Redox interactome dates back to the origins of Life.

**Current Opinion in Physiology** 2019, **9**:34–47

This review comes from a themed issue on **Redox regulation**

Edited by **Sruti Shiva** and **Miriam Cortese-Krott**

For a complete overview see the Issue and the Editorial

Available online 19th April 2019

**https://doi.org/10.1016/j.cophys.2019.04.009**

2468-8673/© 2019 The Author(s). Published by Elsevier Ltd. This is an open access article under the CC BY license (http://creativecommons.org/licenses/by/4.0/).

“The difference between physiology as taught now and in my youth is now the student is given principles; then he was only given facts” — *Sir John Rose Bradford*^4^*, 1928*

“Daß ich erkenne, was die Welt im Innersten zusammenhält. … Wie alles sich zum Ganzen webt, eins in dem Anderen wirkt und lebt!” — *Johann Wolfgang von Goethe, Faust, 1808*

## Introduction and Aim

In this article, we introduce a conceptual framework that offers understanding of how the integration of multidimensional function is achieved. The concept is based upon the nature of electron exchange (Redox) processes that co-evolved with biological complexification, enabling synchronization within a multi-layered system comprising intracellular, intercellular and inter-organ exchange processes essential to the integration of whole-body metabolism.

Physiology is the study of how cells, tissues and organs function within a living system. It embraces the responses to challenges within which the chemical, physical and metabolic processes that enable ongoing functions are modulated. The capability of the body to modify its behavior at every level of organization thereby maintains stability and confers robustness. A cell or organ-based investigative approach enables understanding at those levels, but integration of these individual components within the whole body requires a broader systems-based understanding along the lines adopted by Systems Biology. Systems Biology is a relatively young discipline whose principles are based on the foundation of Ludwig von Bertalanffy’s ‘General Systems Theory’ [1,2]. Bertalanffy strongly objected to reductionism and argued that all systems, whether organic or organizational, are influenced by interaction with their environment and exhibit common characteristics that follow the same underlying principles.

Regulation and control operate at the molecular and subcellular level but the cell is the simplest unit that exhibits the characteristics of independent life. Expanding our understanding and the capability of generating a considerable body of information through the ‘omics revolution’ will provide further detailed knowledge of the many complex interactions [3]. The ordering, structuring, and interpretation of ‘big data’ constitute a substantial, time-consuming task. But even with the information ordered and integrated there remains a major challenge in getting to grips with an adequate understanding of the distinction between inanimate and living matter, between chemistry and biochemistry [4^•^,^5^,6^•^], that limits insight into aspects of fundamental regulatory mechanisms. Much recent emphasis has been placed on considerations around the origin-of-life chemistry and related systems chemistry. Yet, there has been an almost exclusive focus on replication and reproductive aspects with rather less attention being given to those elements or pathways that are essential to survival between episodes of replication; notwithstanding that where there is no survival, there is no replication.

Complementary insights have been derived from computational physiology, the ‘physiome project’ [7] as well as the combination of Systems Biology with Physiology [8] and Evolutionary Biology as well as Philosophy [9^•^,^10^]. One fundamental challenge that remains for Systems Physiology is to identify the organizing principles that enable living systems [11], and which adequately embrace an appreciation of the nature of ‘*robustness*’ and ‘*emergence*’ in Systems Biology, thereby providing meaning to the sense that ‘the whole is greater than the sum of its parts’. There is lack of clarity of meaning for two other terms used in the more recent physiology literature: ‘*resilience*’ and ‘*convergence*’. *Resilience* captures the complex construct that characterizes the ability of an organism, or organization, to withstand and recover from a stressful challenge. The term has been used in contexts as divergent as business, computing, ecology, military combat readiness, and material sciences as well as mental health, physical fitness, and wellness of individuals and populations. An overarching theme in human resilience is the ability to accommodate unforeseen environmental challenge of varying degrees with the potential to recover with return of usual function. This applies in areas as diverse as psychosocial stress, exposure to toxic or biological threat or wider changes in the metabolic needs of the organism. *Convergence* is more straightforward, and there is a growing interest in exploring the relationships between intermediary metabolism, cellular bioenergetics, oxidative stress/Redox signalling, circadian clocks, and inflammation in areas as diverse as immunology, neurodegeneration, cardiovascular function, and cancer [12, 13, 14, 15, 16, 17, 18, 19, 20, 21], with similar developments happening in plant and bacterial research. What triggered this development is unknown. While common underlying principles are often expressed differently from one context to another, there is agreement that many cellular events are closely linked to Redox processes. Thus, these recent insights (and uncertainties) may be considered to be a direct consequence of an increased interest in the concepts around ‘oxidative stress’.

For over three decades, an understanding of ill health has been associated with the oxidative stress concept in the form of ‘an imbalance between oxidants and antioxidants in favor of the oxidants’, which developed into the idea of a ‘disrupted Redox signalling’ [22]. The refinement of this concept has been hastened by the articulation of a ‘Redox Code’ in an influential paper describing a set of principles through which biological function is enabled and protected [23^•^]. Many disease processes are attributed to enhanced production of reactive oxygen species (ROS) or ‘dysfunctional Redox regulation’. Yet, not everything can be explained by excessive ROS production. Many pathophysiological conditions may in fact exemplify processes that can be accounted for by other bioactive entities such as reactive nitrogen or sulfur species (RNS, RSS), or other small signalling molecules such as H_2_, NH_3_, and CO. Many of these entities can react with each other, with protein thiols or other biomolecules. These varied interactions modulate the function of ion channels, enzymes, transcription factors, and other biological targets, a scenario we defined as the ‘Reactive Species Interactome’ [24^•^]. This Reactive Species Interactome concept provides a useful framework to help explain the apparent complexity of adaptive signalling. There is no single marker or process that captures the complexity of these interactions adequately. Rather, it is likely that a combination of readouts from different levels of organization will be required to explain how and why mitochondrial function appears so intimately related to chronic disease, inflammation and metabolism. Being able to distinguish ‘wellbeing’ from ‘feeling ill’ likely will require integrative approaches such as multi-level systems analysis [25, 26, 27, 28].

Systems biology has been proposed as providing new avenues to study metabolic and signalling networks, including Redox metabolism from an integrated systems perspective [29,30^•^]. However, insight may be constrained because of the scarcity of high-quality/high-resolution (spatial and temporal) data across multiple levels of regulation: from cell organelle, through whole body to ecosystem. Moreover, many low-abundance metabolites are often not identified in routine ‘omic analyses. Methodological challenges persist and may not be resolved soon. Limitations with tools for multidimensional mathematical analysis do not readily lend themselves to the identification of the ‘sweet spot’ for non-linear relationships (the Goldilocks principle; [31]), harmonized across the different levels in multi-layered complex life forms/organisms. Nor do they necessarily identify those fundamental functions that are particularly well protected from change.

Rather than reviewing the literature formally, we have sought to offer a view on the pertinent literature by developing an explanation for why and how different physiological functions might be interconnected. We approached this from a consideration that provides a rational perspective on Redox-mediated or modulated events and offers a perspective that can be modelled and integrated with ongoing analytical methods and approaches. To clarify how these physiological functions have developed and are related it is necessary to reflect on the evolutionary journey required for coping with environmental challenge since the beginnings of Life. This reflection indicates that the Redox architecture of physiological function that we see today has its origin in the processes that enabled all forms of life to emerge and cope with the exigencies of day-to-day life in changing, and sometimes hostile, environmental conditions. This is captured as the underlying principle for the generic response for survival. We have chosen examples from the nitric oxide (NO) field of research to illustrate the concept, not only because it is a ubiquitous signalling molecule whose production and metabolism is dependent on cellular Redox status, but also because it acts as a chain-breaking antioxidant [32] and even its prototypical receptor (soluble guanylate cyclase) appears to be under Redox control [33]. We sought to avoid any particular oxygen/ROS-centric perspective, which can limit consideration of other relationships of similar or even greater importance, in order to place emphasis and greater clarity on the more general term ‘Redox stress’.

## Redox as life’s ‘Lingua Franca’ – how it all began

### Redox landscape and Redox interactome

**Scheme 1:**
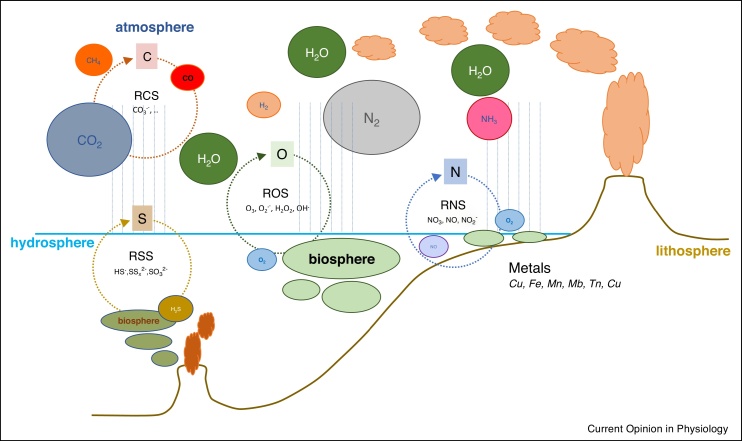
The Redox landscape on Earth. The Redox landscape of the biosphere (green ellipses) is connected to the inorganic chemistry of the atmosphere, the oceans, and the earth mantle through interactions among Reactive Nitrogen, Carbon, Oxygen and Sulfur Species.

If chemistry provides the foundation upon which biomolecules interact, Redox chemistry constitutes the universal language that enables communication between different entities and layers of regulation. By definition all biomolecules can engage in ‘*Redox*’ (i.e. electron transfer) processes because their atomic constituents can either act as electron donors (**red**uctants) or acceptors (**ox**idants). The Redox activity will depend on the chemical properties of the set of molecules with which they interact, that is, their Redox environment [34]. The potential effective environment is extensive (Scheme 1). The simplest Redox molecules consist of small gases such as H_2_, H_2_S, NO, O_2_, CO_2_ and others as well as metal ions such as iron, copper, molybdenum and tungsten [35,36]. Most reviews dealing with oxidative stress/Redox biology place emphasis on ROS (including superoxide and hydrogen peroxide) and thiol compounds (such as glutathione or thioredoxin). However, these constitute only a small proportion of the potential Redox population that includes many S (and Se), N or C-based Redox active compounds. Although focused scientific enquiry has tended to place emphasis on select compartments, in nature all Redox molecules may cross-react, forming a single synchronized network of electron-exchanging entities.

In order to exert their effect gaseous compounds do not require specialized machinery for transport or sensing. Indeed, they can exert influence beyond recognized borders (i.e. extending from solid matter to the atmosphere). They can diffuse and react over long distances, thereby extending the range of Redox interactions. Redox chemistry embraces chains of reactions that extend beyond the first Redox couple with each reaction having the potential to generate new Redox-active molecule(s) that in turn react(s) with many others. The reaction network might expand in time and space, generating a system of interconnected, dynamic interactions that has been referred to as the *Redox Landscape* [37]. Here, we consider a boundary-limited entity, the mammalian body.

#### Disambiguation 1. The fleeting identity of reactive Redox species

In Redox chemistry, the notion of Identity (symbolized by a name and a structure) is a dynamic concept. Redox molecules are not stable and formally exist only for a certain, often short, period of time [34,38,39]. For example, the lifetime of nitric oxide (NO) in blood is in the order of milliseconds whereas low NO concentrations in the atmosphere can persist for hours. As Redox molecules are continuously interacting with each other, rather than seeking to identify individual Redox molecules it is more appropriate to consider the *Redox Interactome* as an entity in its own right: a pattern of Redox species whose particular character is determined by considerations related to history and ecological context.

### Emergence of Redox biology

As Life emerged, available metals, gases, and small molecules including S, N, and C-based compounds became intimately involved in multiple Redox interactions. Redox chemistry is the basis for the interdependence between living entities and their local environment, the bridge between inanimate and living matter, between the biosphere, the hydrosphere, and the atmosphere [40] (Scheme 1). The strong Redox transformations within the first two billion years of Earth’s existence [41,42] will have been a major driver for Redox biology. In a reducing environment, the ‘oxidative power’ of living systems may have been an important driving force as opposed to the ‘reducing power’ in contemporary life forms, with antireductants rather than antioxidants essential to protecting cellular integrity.

Life can be seen as a network of metabolic processes that show the capability to maintain themselves under changing environmental conditions. This is illustrated by the ‘Great Oxidation Event’ [43]. The photosynthetic production of O_2_ created an oxidative burst in an environment that previously had been largely reducing. Simple catalyst such as NO might have played a critical role in enabling organisms to cope with potentially toxic oxygen-related compounds [44,45]. This would represent a major Redox revolution that transformed Earth’s environment and modified the *Redox landscape* for living entities [46,47] wherein the emergence of novel Redox molecules and interactions shaped the history of Life as we know it. Thus, Redox may have been the first language in the history of living matter.

## Evolution of Redox agents

### Biomolecules increase in complexity of form and chemistry

**Scheme 2:**
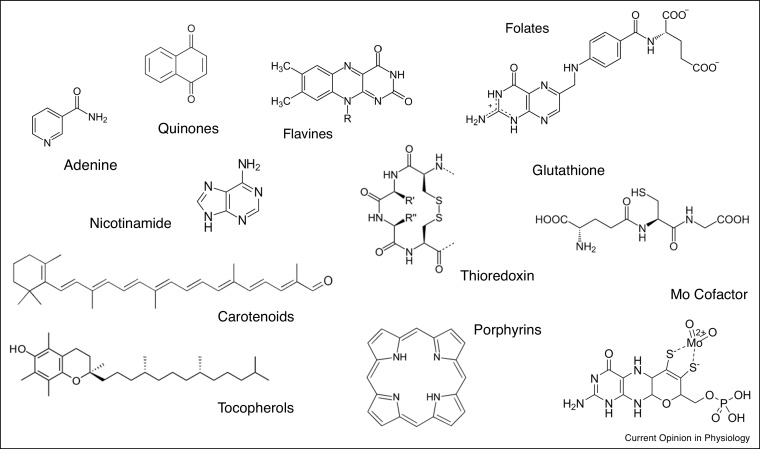
Increasing complexity of major Redox-active biomolecules. Examples of contemporary Redox cofactors displaying various types of Redox reactivity (heterocycles, aromatics, unsaturated bonds, thiols, metallo-complexes etc.).

As life forms diversified and became more complex, new entities emerged. In addition to ROS, RNS, and RSS, other carbon-based biomolecules with enhanced Redox power emerged, for example conjugated unsaturated bonds or unpaired electrons, and metal complexes. Examples include tocopherols, carotenoids, polyphenols, porphyrins, ascorbate, glutathione, and folate (Scheme 2). These more complex structures afforded greater stability, and although their Redox properties might have been more constrained their complex structure provided an advantage by enhancing network stability. Interactions amongst these simple Redox molecules correspond to basic, first-order chemistry governed by classical thermodynamics. A new dimension was introduced to this Redox space through the emergence of *Redox catalysis*.

### The emergence of Redox catalysis

At some stage, metals and other Redox centers became associated with polypeptides, forming new kinds of Redox agents characterized by a rigid scaffold that enabled the achievement of more selective chemistry that would be especially useful for specific biological functions. Moreover, these Redox machineries enabled catalysis of reactions that could not have taken place otherwise, generating a more diverse and fecund *Redox landscape*.

There are many known Redox proteins that appear to have been conserved from the early stages of Life, such as metallo-proteins and proteins associated with Redox cofactors [48] (Scheme 2). They show an extensive range of global architectures and fine structures, illustrating the power of evolution in enabling a rich and profuse Redox language [49]. Thus, the range of Redox chemistry evolved in parallel with biological evolution, enriching the Redox language with many new possibilities.

#### Disambiguation 2. The versatility of Redox catalysis: a name is not a function

Novel structures are the material of evolution. The structure of Redox proteins has been modulated during the course of evolution giving rise to a large array of distinct isoforms that emerged alongside environmental changes, for example, cytochrome P_450_, NOS, SOD, and others [50,51]. It is, therefore, not possible to assign a specific Redox chemistry to any particular family of proteins. The versatility of Redox chemistry is related to structural modifications, environmental changes and their combination. To illustrate, the catalytic site of nitric oxide synthase (NOS) is a hub for a multitude of Redox chemistries [52]. As an oxidase, it achieves a double oxidation of a single substrate (l-arginine) into NO and citrulline [53]. However, it can also produce superoxide, hydrogen peroxide and peroxynitrite under particular conditions [54]. The very same heme-thiolate moiety also entertains alternative chemistries such as the reduction of nitrite, homolytic cleavage of O—O bonds, dioxygenation of NO, peroxidation of nitrite, among other chemistries [55]. The ability to achieve a range of Redox reactions that are closely related to their environment contributes to the dynamic complexity of the *Redox Interactome*.

### Increasingly sophisticated architectures

**Scheme 3:**
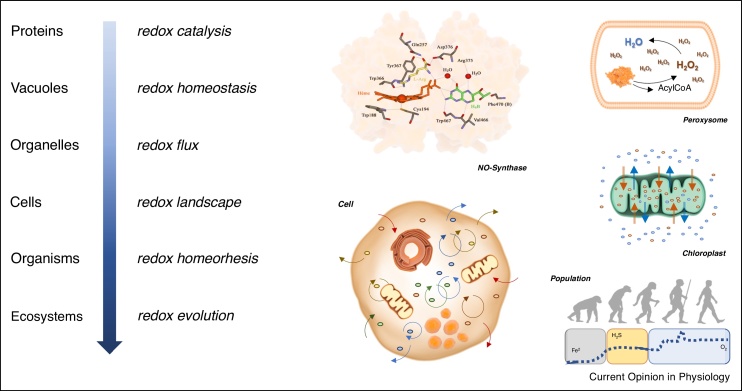
Complexification and stabilizing Redox machineries. Examples of increasingly complex Redox systems showing compartmentalization and synchronization of different Redox biochemistries, including the catalytic pocket of a protein, the isolated oxidative chemistry of a peroxisome, the build-up of transmembrane electrochemical gradients in a chloroplast, the intracellular coupling of Redox processes within a cell, and the variations in Redox environments coupled to organismal evolution.

With time, living entities acquired increasingly more complex subcellular structures encompassing additional motifs including membranes, vesicles, vacuoles, and organelles (Scheme 3). Compartmentalization offers a powerful way to exploit the specific potential of particular aspects of Redox chemistry [56]. This is exemplified *par excellence* by the structure and function of the electron transport chain. This incredibly sophisticated system of Redox entities (proteins, metals, iron–sulfur clusters, cofactors etc.) facilitates a complex chain of reactions that together enable the generation of an electrochemical (Redox) gradient [57]. The ability to achieve this Redox gradient sits at the heart of Life’s bioenergetics.

As Life increased in complexity from cells to tissues and organisms, communities and ecosystems, its internal communication system (***Redox*** as both *lingua franca* and *mechanism*) evolved in parallel. The same *Redox landscape* extended to greater dimensions, maintaining its structure in the form of a dense and interconnected network. Most cellular life forms are embedded within a common Redox environment, remain continuously interconnected within a single Redox system, and share the same *Redox landscape*.

## Evolution of Redox function

Through evolutionary complexification of form and chemistry the Redox network acquired novel functional capabilities (Scheme 3). To push the linguistic metaphor, the Redox language was enriched not only by the invention of new words but also by the development of new rules of grammar, allowing the formation of new sentences and definition of new processes and functions [58].

### Stress, antioxidant Defense and Homeorhesis

An early first imperative for interacting Redox molecules was probably the establishment and maintenance of a form of Redox equilibrium within an ever-changing Redox environment. The emergence of antireductants [59] and antioxidants [45] would be required to provide the first line of defense and to counter reductive or oxidative stress.

#### Disambiguation 3. Stress, antioxidants, and Homeostasis

Redox Biology is not limited to the field of Stress. *Redox Stress* is widely considered to represent indiscriminate, uncontrolled, extensive and intense production of oxidative or reductive molecules. The current perception is that living organisms need to cope with such challenges by mobilizing an *Antioxidant Defense* and sustaining a *Redox Buffer* to preserve *Redox Homeostasis*. Yet, this notion ignores the fundamentals of Redox biochemistry. These terms refer to relatively static relationships within a system that aims to keep cellular and organelle Redox potentials (often mistaken to reflect ROS concentrations or GSH/GSSG ratio) within a certain range by eliminating excess reductants or oxidants in order to achieve and/or maintain a better balance. This fails to account for the fact that Redox reactions imply differences in Redox potential (and not quantity) among a multiplicity of reactants some of which have the tendency to diffuse away or be transported elsewhere. In reality, it is not possible for such a thing as *Redox Equilibrium* or Redox *Homeostasis* to exist in an open, dynamic and ever-changing biological milieu.

A more appropriate conceptualization is that living entities accommodate their Redox environment, not the other way round. The concept of *Homeorhesis* [60], introduced by Conrad Waddington in the 1940s, acknowledges the impossibility of a stable, invariable equilibrium. Instead, it suggests a dynamic process that preferentially selects living entities capable of employing novel Redox chemistry and biochemical processes in response to environmental perturbations (a concept that extends to the adenylate-based bioenergetic system of living cells [61]). ‘Redox Engineering’ recognizes the preservation of the Redox status in which live forms unfolded. However, it also acknowledges that a modified Redox landscape may represent both a potential threat on the one hand, and a driving force offering new opportunities to be taken advantage of, on the other.

### Homeostasis versus Signaling

Redox Biology is often presented as a balancing act between *Redox Homeostasis* and *Redox Signaling*, with both considered to represent parallel, unrelated processes. A transition from *Redox Buffering* to *Signaling* mode is invoked, but nowhere is the process detailed or explained. At most, the concept of a signal is depicted in the form of ‘excess production of ROS’ overriding the antioxidant defense [62, 63, 64]. However, in real life these Redox activities must be coupled because the same Redox agents engage simultaneously in both processes.

In a similar way to NO [65,66], the same Redox molecules might be engaged in distinct and unrelated metabolic processes at the same time. It has been proposed that the nature and extent of RNS production, depending on the condition of the milieu in which it is generated, might determine the nature of the biological effect (stress versus signal) and the type of signaling [67].

All Redox processes rely on the same network of Redox species, continuously interacting with each other. This leads to the simultaneous superimposition of multiple activities that remain interconnected through a common *Redox Interactome* the nature of which is determined by the physicochemical environment. Consequently, Redox chemistry cannot be limited to a simplistic homeostasis/signal balance as it pervades all metabolic and functional reactions. A complete review of the diversity of Redox Physiology would be necessary to describe all physiological processes that are potentially linked, one way or another, to Redox chemistry. Below we introduce just a few examples.

### Symbiosis, Immunity

Because of its universal character, Redox chemistry has been extensively considered to represent a mechanism for inter-cellular signaling [23^•^,^67^,^68^]. This is illustrated by NO which has been considered to be an archetypal mediator [69,70]. NO is central to the initiation and regulation of the symbiosis of plants/bacteria [71], corals/bacteria [72], cephalopods/bacteria [73], and other microbial symbioses [74]. This symbiotic tuning is related to signaling processes mediated by nitrosylation or nitrosation-mediated processes. However, under oxidative conditions, NO is rapidly converted into other (sometimes more toxic) RNSs. As the concentrations of oxygen in the oceans rise (creating a more oxidative environment), all forms of NO gradually produce ‘nitrosative stress’ which is cytotoxic for marine life. What was first used as a symbiotic signal will eventually become an ‘Eviction Notice’ [75,76]. In the wake of this new *Redox landscape*, non-specific immune reactions emerged as new types of Redox activity. Similar processes are likely at play in mammalian cells and tissues under inflammatory conditions (known to be associated with oxidative stress) affecting innate immunity. In addition, NO/ROS has been shown to affect immunometabolism by modulating mitochondrial function.

In all of the above, an extensive population of different Redox agents with distinct chemistries simultaneously engages in various, often opposite, functions as a result of its Redox environment. The simultaneity of functions is well illustrated in the symbiotic relationship between the bobtail squid (*Euprymna scolopes*) and a bioluminescent bacterium (*Vibrio fischerii*). Here, NO is involved at the same time in squid immunity (preventing the infection from various microbes) and in the initiation of a selective symbiotic relationship with a unique species of bacteria [77]. These examples of pleiotropy seem to be a consequence of co-evolution of organisms and their environment, and the exaptation (i.e. the process by which certain features acquire functions for which they were not originally selected) and adaptation of structure to new Redox landscapes [78].

### Detoxification, catabolism

Secure metabolism of xenobiotics is fundamental to human health and may be considered to be a remnant of a primeval Redox defense. Redox chemistry is the first, essential step for the complex detoxification processes achieved by cytochrome P_450_ enzymes, a large family of proteins with >300 000 isoforms across the Tree of Life. It consists of the oxidation (e.g. hydroxylation, epoxidation) of a multitude of xenobiotics aimed at limiting their potential toxicity and facilitating their excretion. In contemporary bacteria, plants, and mammalian species, however, cytochrome P_450_ enzymes also enable the biosynthesis of a large number of new compounds, contributing for example to sterol and lignin production.

### Bioenergetics, Metabolism

The most impressive exemplars of Redox chemistry are, without doubt, the Electron Transport Chains (ETC), which integrate many different types of Redox molecules to achieve a complex outcome. In the thylakoid membrane, the ETC catalyzes the splitting of water, oxygen degassing and carbohydrate biosynthesis, whereas in bacteria, it enables coupling of substrate metabolism to transporter fluxes and environmental sensors and provides energy for growth. In the inner membrane of mitochondria, the ETC promotes the build-up of the proton and electrochemical gradient necessary for ATP production. Several Redox reactions and molecules, usually coupled to downstream electron acceptors, can lead to ROS production when electrons are backing up in the chain. This can lead to retrograde signaling in chloroplasts [79], for example causing the oxidative stress responsible for coral bleaching [80]. The ETC is a prime example of the connection of bioenergetic pathways to all other Redox processes through the *Redox Interactome*. As bioenergetic regulation is at the intersection of all metabolic pathways, Redox processes are linked to most if not all physiological processes.

Life is dynamic. Unlike inanimate matter, the chemistry of living entities operates at far-from-equilibrium conditions, escaping the 2^nd^ Law of Thermodynamics [4^•^]. To sustain Life requires the continuous provision of energy. This permanent effort against entropy is enabled by a ceaseless energy flux. It is what transforms matter into living entities as dynamic and evolving systems. The ‘out-of-equilibrium’ status enables or requires a continuous change of the living structures and processes leading to a progressive complexification of living systems [81]. This is where Redox chemistry, the *Lingua Franca* of metabolic and bioenergetic processes, plays its most fundamental role.

## Stabilization and synchronization

Organisms face a paradoxical double injunction. Whereas the Redox processes remain deeply interconnected and synchronized within an organism (all Redox agents diffuse and cross-react with each other), at the same time they must allow selective Redox chemistry to take place at specific cellular locations. To prevent the Redox language from becoming incoherent, organizational principles must have emerged at an early juncture of Life’s evolution through which it became possible to streamline the wealth of interacting words/biomolecules, sentences/structures and meanings/functions. There are different options through which it is possible to organize, isolate and stabilize aspects of Redox relationships, four of which are considered here.

### Localization

*Redox Signaling* requires the use of a sufficiently stable and specific agent, tightly controlled production, and a specific target within a constrained space. This is illustrated by the concept of pulsed enzymology [82], which characterizes the operation of NO in neuronal signaling [83]. By contrast, uncontrolled production or release of Redox-active molecules would risk interfering with local Redox processes by acting in an untargeted fashion at unintended locations. In skeletal muscle, for example, Redox processes play an important role in generating force [84]; here, antioxidants have been shown to prevent the health-promoting effect of physical exercise [85] by interfering with oxidant signaling. Similar interference with other Redox processes might explain the unexpected observation of an increased mortality associated with supplemental antioxidant therapy [86,87].

Alternatively, certain Redox functions may be protected through cellular compartmentalization. The development of organelles and other cellular structures permits the formation of inner micro-environments that enable specialization of particular Redox chemistries, as exemplified by the ETC. Here, most of the Redox processes are organized on membrane scaffolds with an extremely sophisticated architecture, allowing the partitioning of charges and the build-up of proton gradients. At a higher level, nitrogen fixation requires specific settings that are only found in certain types of cells (heterocyst, nodules [88,89]). Examples of specific cellular architecture are hydrogenase activity which requires an anoxic environment as found in the hydrogenosome, compared with proper protein folding which requires the oxidative environment provided by the endoplasmic reticulum [90].

### Specialization

Thus, structural complexification afforded confinement of specific Redox chemistries and specialized molecules for use in select functions. While this may seem obvious for metallo-proteins (whose biochemistry is constrained by the second coordination sphere interaction between heme-bound oxygen and the distal environment of the heme), it has also been proposed for simpler Redox molecules. ‘The Good, the Bad and the Ugly’ paradigm was proposed for NO and related RNS. Here, a specific Redox activity (nitrosylation) might be utilized for signaling functions, while other more reactive species such as peroxynitrite (ONOO^−^) might be associated with cytotoxic activities [67]. To ensure selectivity, possible side-reactions must be minimized. This could best be achieved by relying on short-distance, rapid reactions where possible. The concept of kinetic specificity [37], might offer an explanation for the varied although highly selective reactions of RNSs and RSSs. Ultra-rapid reactions allow the use of low concentrations and constrain the signal to shorter distances. In this regard, Redox proteins would have offered a solution by increasing reaction rates and thereby limiting reaction space.

### Redox bandwidth

Each molecule has a specific Redox potential (determined by measurement against a reference electrode) that determines its reactivity with other molecules. A reaction between two molecules will rely on the difference between their Redox potential, and the fate of a molecule will reflect the kinetic competition between several possible reactions. In theory, this should allow the development of ‘quantitative Redox biology’ [91]. However, in biology these considerations are complicated by the fact that mutual interactions are highly influenced by compartmentalization and specific catalysis. Nevertheless, abundant weakly reducing/oxidizing entities will be universal Redox scavengers with low specificity whereas short-lived but highly reducing/oxidizing Redox agents will engage in specific signals. It is likely that different Redox ranges (bandwidth) are associated with specific biological functions and/or are required for different cells (or sensing/adjustment within specific locations) to cope with normal physiological stressors. Conceivably, a shift in intra/extracellular Redox status will impact all local Redox processes by limiting the possible Redox bandwidth in that environment, irrespective of its effects on bioenergetics.

### Synchronization and Integration

**Scheme 4:**
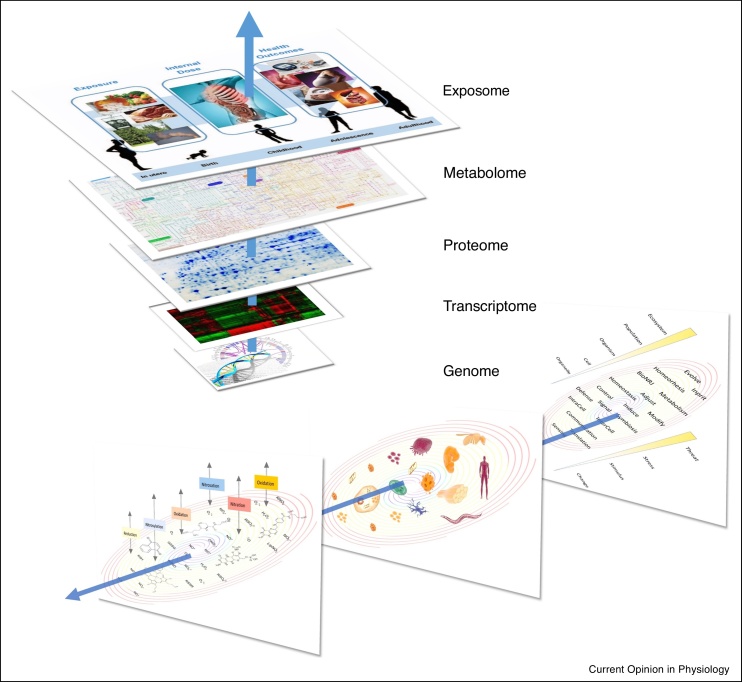
Orthogonality of Redox regulation across levels of biological organization. *Upper panel:* The Redox Spine connecting all layers of regulation. *Lower panel:* 1. Multiplicity and evolution of Redox agents; arrows reflect the various types of Redox chemistry as a function of time/environment. 2. The complexification of Redox structure as a function of time, from biomolecules to organisms. 3. Evolution and diversification of Redox function, from sensing to adaptation.

As all Redox reactions converge toward a single purpose (i.e. maintaining the integrity of the individual cell or organism), all these different levels of regulation are integrated into one single architecture. The synchronization of all processes is made possible by the specific properties of Redox chemistry: all Redox molecules, all components and compartments, all processes and functions are interconnected through a coherent ***lingua franca*** in a network of interactions unfolding in space and time. This ‘*Redox spine*’ connects all levels of regulation (Scheme 4), allowing the opportunity for tuning into a single and harmonic partition of all successive levels of complexity, from the labile S-nitroso moiety of a specific protein thiol to the tissue, from simple enzymatic reaction in a specific compartment to whole-body energy status and metabolic regulation.

## Adjusting, adapting, surviving

Even highly compartmentalized *Redox landscapes* of individual organisms are open and dynamic, and carry the possibility for divergence in the face of particular challenges from the environment. Conditions that predispose to pathology might thus be regarded as those where a loss of a stable specialization results in local or global desynchronization of Redox processes leading to abnormal function against the background of ongoing oxidative or reductive challenges.

*Stress* is a necessary response to changes in demand or environmental conditions [92] that needs to be considered always in the context of the biological level at which it takes place: protein, organelle, cell, organism, population, ecosystem. Whereas the subtle oxidative modification of a particular protein/metabolite might suffice for cellular accommodation under one circumstance, sacrificing an entire species may be required to allow survival of an ecosystem in another situation. Likewise, oxidative/reductive modification may be deleterious at a specific level of organization but not for the entire organism. In this regard, *Redox stress* is not necessarily bad (hormesis, adaptive homeostasis [93,94]). The question as to whether certain Redox changes are beneficial or deleterious depends on the level of organization, from cell to ecosystem. Physical exercise can be considered an example of a physiological stressor associated with enhanced oxidant production that is beneficial for human health by stimulating downstream Nrf2-related antioxidant defense systems [95].

Physiological reactions to stress differ depending on the biological level affected. Exposed to acute Redox stress, cells respond by adjusting metabolism to preserve structural and functional integrity, a process resulting in a modification of the *Redox landscape* (enhanced production of glutathione and heme, protecting free thiols, diminishing ROS impact, etc.). The outcome (survival, apoptosis, cell death) will depend on the available bioenergetic situation and adequacy of precursor supply to support the metabolic changes. During short-lasting high-intensity exercise (e.g. an athlete running a 400 m race) when the muscle is generating maximal forces, there is an instantaneous increase in ATP utilization that places an extreme local demand on cellular bioenergetics with resultant changes in Redox state to maintain ATP resynthesis. Cell death can only be avoided by ensuring that the metabolic consequences of hypoxia and acidosis within the motor unit that generates force is accommodated through concomitant changes in Redox state within the non-recruited motor units of the same muscle as well as across other tissues (e.g. liver and lung) and body fluids (buffering through changes in base excess).

This coordinated multi-level collective response enables the local demands to be met whilst mitigating the potentially damaging effects across the body as a whole. By comparison, facing a more global, longer-lasting stress, an organism will survive by utilizing ‘long-range signaling’ (‘systemic acquired resistance’ in plants) by modifying its functioning and adjusting its metabolic parameters to sustain transient but extensive Redox adjustment. An example for this situation is the inescapable hypobaric hypoxia experienced by those who sojourn to high altitude. Environmental hypoxia is associated with enhanced oxidative stress with compensatorily increased production of NO and adjustment in the handling of many other metabolites [96,97]. By contrast, a population existing at altitude has to acquire Redox processes and chronic functional change that enable ongoing function without potential jeopardy to daily life, work or reproductive success. In this situation, genetic and epigenetic variability will facilitate the emergence of ways to improve survival and reproductive success in the different environment. Tibetan highlanders are an example of a population living at high-altitude who have evolved adaptations to the challenges of life in chronic hypoxia. Their whole-body production of NO is upregulated [98,99], and also adjustments have taken place at numerous other levels (including genetic and metabolic) within a surprisingly short period of time (10–20 millenia) indicative of the strong Darwinian selection pressure experienced [97, 98, 99, 100].

Thus, in answering the question of how to cope with *Redox stress* requires consideration of the scale and type of interaction with the environment. Whereas the cell might adjust its inner milieu (Redox landscape) to preserve its integrity, the adjustment for a population might be observed at the whole-body or group level. Although each biological entity might trigger a different response to the hypoxic stress, all these responses will be based on the same Redox language and integrated coherently to enhance resilience.

### Disambiguation 4. Beyond good and evil

Dobzhansky’s notion that “Nothing in Biology Makes Sense Except in the Light of Evolution” [101] places emphasis on the inadequacy of a conceptual model which simply considers biology as an achieved state of equilibrium and stability. Similar conceptual constraints apply to Redox biology. Structures and processes have been assigned roles attributed to preservation of the organism’s *Redox homeostasis*, whereas changes in the *Redox landscape* are considered as a stress to be countered. This concept fails to take adequate account of the potential richness of Redox physiology. Such Manichean vision is also at odds with the progressively acquired capabilities that characterize evolutionary development [4^•^,9^•^]. The Redox landscape is a continuum of Redox reactions that are tightly connected to a physico-chemical environment in permanent evolution. Dynamic Redox changes opened a new and wide register of possible interactions that act as the basis for dealing with variability and driving evolution. In the bigger picture, the long-term benefits of novel Redox interactions may more than offset the disadvantages associated with a measure of collateral damage.

Put concisely, *Redox Stress* enables changes to take place that override the existing mechanisms which evolved to conserve and preserve the *status quo*. In fact, it is more likely to be a driving force for complexification. *Stress* provides new *Redox landscapes*, in which each living entity is driven to adjust their metabolic machinery to enable new relations in Redox chemistry linked to the emergence of novel biological processes. These Redox interactions, essential for the development of Life on Earth (especially during the ‘Great Oxidation Event’), could be considered to have been a major evolutionary driving force.

## Nutritional considerations

From a nutritional perspective, it is important to ensure that the internal metabolic environment of the body is maintained in the face of marked variations in the external environment. An ongoing exogenous supply of substrates and co-factors (from diet and microbiome) is imperative for Life, and the ability to cope with the metabolic demands elicited by a range of stressors (activity and/or pathology) is paramount. Good health can be secured only when the physiological and metabolic demands of cells, tissues and organs are continually met with an adequate reserve to buffer periods of uncertainty. This inevitably requires sufficient metabolic plasticity to accommodate unusual stresses or changes in supply. Failing to meet those demands will result in accommodations in structure and function to maintain life at the cost of diminished reserves and greater vulnerability. Modest deficits in the short-term may be accommodated by altering metabolic priorities across the whole system, albeit with accumulating deficits over time. With ongoing challenge, the adaptations required to sustain Life will ultimately be overwhelmed. The consequence will be a resultant loss of metabolic control and an inability to maintain the constancy of the internal environment. It is this inability to protect the internal environment that ultimately is expressed as increasing ill-health, morbidity and mortality.

Thus, a central feature of health is the ability to maintain the metabolic machinery that enables cellular bioenergetics and Redox state throughout the body. The sensing and adaptive signalling systems operating amongst cells and tissues have to interact collectively in order to maintain the internal milieu of the system as a whole. Although the diet may ultimately provide sufficient energy and a pattern of nutrients to support the system as a whole, there is the need to consider how the specific requirements of individual cells and tissues are met within a dynamic system of exchange and interchange. The demands for specific nutrients to maintain usual structure and function together with the capability to respond effectively to external stressors has to accommodate potentially competing demands in different compartments over time. This consideration refocuses attention on those nutrients that are known to serve as cofactors and essential determinants of mitochondrial metabolic function: such as the B vitamins (especially thiamine, riboflavin and pyridoxine) or choline (for lipid synthesis), together with metals such as iron, zinc and copper and appropriate sources for sulfur and nitrogen-based compounds. At this level, the importance of recently recognized metastable species such as polysulfides (compounds comprising chains of sulfur) have to be considered [102,103^•^]. Their particular roles in mitochondrial function/cellular energetics and whole-body Redox regulation require elucidation. Mitochondrial dysfunction with altered cellular Redox status is a common feature across many medical conditions. Targeted provision of nutrients, exemplified by ‘feeding the mitochondria’ [104,105], may mitigate adverse change and lead to improvements in structure and function. Further, the availability of conditionally essential nutrients (such as the amino acids glutamine, serine and glycine) together with folate (as a methyl carrier) and their interactions with methionine metabolism critically determine the maintenance of intracellular and systemic glutathione, especially in the stressed state. Such considerations have to be embraced within a more holistic approach to nutrition and Redox biology in which the *Redox interactome* represents the responsive interface between the individual and its environment [106^•^]. Clearly, delivering ‘precision nutrition’ is not without challenges [107].

## Future challenges and opportunities

Confusion in the Redox literature can arise from a lack of clarity in definition and terminology. There is the use of non-specific terms or concepts that do not obviously conform with fundamental principles of biology. Imprecision may be due to ambiguity (being open to multiple interpretations – e.g. Redox buffer), vagueness (the bound of its extension is indeterminate – at what point, for example, does a Redox state become ‘unfavourable’?) or equivocation (to avoid committing to a true meaning – e.g. ‘Redox landscape’). The lack of consensus on the precise meaning of any given word, phrase or term allows for differences in interpretation. This leads to confusion in practice as the same term is either used differently in different contexts, or different terms are used to describe the same or similar processes. Thus, progress in Redox Physiology is constrained not only by analytical methodology and the appropriate application of statistical or conceptual models, but also by the need for a clarity of thought and definition that ensures that the understanding derived from the processes under study can be communicated with fidelity.

We can now be certain that by simply measuring the concentration of a single and readily accessible Redox metabolite it is not possible to adequately characterize the dynamic nature of a multi-level system. Changes in one compartment likely result in both increases and/or decreases in Redox state in other compartments so that overall Redox state of the organism as a whole is maintained. How this is achieved remains poorly understood. One approach to improving our understanding of the *Redox landscape* may be to adopt a dashboard ‘omic-style approach using concurrent measurements of multiple aspects of Redox state at different levels of the system in a dynamic fashion (e.g. at rest and during exercise, fasting versus fed, normoxia versus hypoxia, etc.). This approach requires moving beyond measures that capture a static perspective of the system to the determination of kinetic measures of flux through a limited number of key nutrient-sensitive metabolic pathways (e.g. ascorbate and GSH production, arginine with its metabolites and determinants of one-carbon metabolism). The measurement of the availability of key micronutrients in functional pools and the analysis of the data using a multi-level holistic systems biology approach will be necessary to adequately model Redox state at the level of the cell, tissue, organ and whole body. Welcome back to the future of physiology!

## Conflict of interest statement

Nothing declared.

## References and recommended reading

Papers of particular interest, published within the period of review, have been highlighted as:

• of special interest

## Footnotes

4 Cited in the Preface of Barcoft, J: Features in the architecture of physiological function. 1938; Cambridge University Press, London, UK; 368 pages.
